# Palmitoylethanolamide and Luteolin in Brain Aging and Cognitive Decline: Biological Rationale and Current Evidence

**DOI:** 10.3390/nu18182949

**Published:** 2026-09-09

**Authors:** Francesca Mancinetti, Virginia Boccardi, Marta Valenza, Martina Alunno, Michela Scamosci, Roberta Facchinetti, Veronica Tecchio, Anna Giulia Guazzarini, Martina Procaccini, Luigi Cari, Giuseppe Nocentini, Carmelinda Ruggiero, Caterina Scuderi, Luca Steardo, Patrizia Mecocci

**Affiliations:** 1 Division of Gerontology and Geriatrics, Department of Medicine and Surgery, University of Perugia, 06129 Perugia, Italy; 2 Department of Physiology and Pharmacology “Vittorio Erspamer”, Sapienza University of Rome, 00185 Roma, Italy; 3 Department of Life Sciences, Health and Health Professions, Link Campus University, 00165 Roma, Italy; 4 Division of Pharmacology, Department of Medicine and Surgery, University of Perugia, 06129 Perugia, Italy; 5 Division of Clinical Geriatrics, Department of Neurobiology, Care Sciences and Society, Karolinska Institutet, 171 77 Stockholm, Sweden

**Keywords:** aging, cognitive decline, cognitive impairment, luteolin, neuroinflammation, palmitoylethanolamide

## Abstract

Cognitive decline is closely associated with biological aging and with interconnected processes, including chronic low-grade inflammation, oxidative stress, mitochondrial dysfunction, and cellular senescence. Nutraceutical strategies capable of modulating several of these pathways may represent a complementary approach to supporting cognitive health. This narrative review examines the biological rationale and available evidence regarding palmitoylethanolamide (PEA), luteolin, and their co-ultramicronized formulation (PEA-Lut) in cognitive impairment and dementia. Evidence from mechanistic studies, animal models, observational studies, and clinical trials was considered. PEA is an endogenous N-acylethanolamine with anti-inflammatory and neuroprotective properties, whereas luteolin exerts complementary antioxidant and anti-inflammatory effects. Co-ultramicronization may improve their physicochemical properties and biological activity. Preclinical studies suggest that PEA-Lut may attenuate glial activation, pro-inflammatory signaling, oxidative and nitrosative stress, and amyloid-β-induced cellular injury while supporting neurotrophic signaling and neuronal survival. Preliminary clinical findings have suggested possible benefits in stroke rehabilitation, frontotemporal dementia, and other neuroinflammatory conditions. However, human evidence specifically addressing mild cognitive impairment and Alzheimer’s disease remains limited, heterogeneous, and largely derived from preclinical and small or non-randomized studies. PEA-Lut therefore represents a biologically plausible investigational nutraceutical approach, although available clinical safety data are limited to relatively small and heterogeneous populations and short observation periods. Adequately powered randomized controlled trials are needed to determine its clinical efficacy, optimal timing and dosage, and long-term safety in individuals at risk of dementia.

## 1. Introduction

Aging is defined by a complex set of structural and functional changes affecting multiple organs and physiological systems. Over time, the ability to maintain homeostasis and respond to environmental challenges declines, resulting in increased vulnerability to disease and death. Although some degree of change in physical and cognitive function may accompany aging, clinically relevant cognitive decline is not an inevitable consequence of this process. In some individuals, however, cognitive impairment may progress to dementia, most commonly due to Alzheimer’s disease (AD) [1]. Dementia is characterized by deterioration in memory and other cognitive abilities that interferes with independence in everyday activities, posing substantial challenges for affected individuals, their families, and the healthcare system. AD can be conceptualized as a clinical and biological continuum, extending from a preclinical phase to mild cognitive impairment (MCI) due to AD and, subsequently, AD dementia of increasing severity [2].

MCI represents an intermediate clinical state between normal cognition and dementia [3]. It is characterized by subjective and objective evidence of cognitive decline beyond that expected for an individual’s age and educational level, without substantial impairment of independence in everyday activities. Individuals with MCI are at increased risk of developing dementia, particularly AD. However, progression is not inevitable, and cognitive status may remain stable or, in some cases, improve [4]. The clinical diagnosis of AD dementia requires cognitive impairment that interferes with daily functioning and cannot be more appropriately explained by another neurological, psychiatric, or systemic condition [5]. From a neuropathological perspective, in AD, there is evidence of the accumulation of amyloid-β-containing plaques and neurofibrillary tangles composed of hyperphosphorylated tau. These alterations are associated with synaptic dysfunction, neuronal loss, and disruption of hippocampal and cortical networks involved in memory and other cognitive functions, leading to poor consolidation of short-term memory into long-term memory [6,7].

Estimates of annual progression from MCI to dementia vary according to the population studied, clinical setting, follow-up duration, and presence of AD biomarkers [8]. Early identification and appropriate management of potentially modifiable factors may nevertheless provide an opportunity to support cognitive health and delay functional decline. Although dementia does not affect all older adults, its incidence and prevalence increase substantially with age [9].

The global number of people living with dementia has been projected to rise from approximately 57.4 million in 2019 to 152.8 million by 2050 [10].

Several interrelated mechanisms may contribute to the association between aging and dementia, including chronic inflammation [11,12], oxidative stress [12], alterations in neurotransmitter metabolism [13], mitochondrial dysfunction, and cellular senescence. Available symptomatic treatments provide limited clinical benefits, while emerging disease-modifying therapies may slow cognitive and functional decline in selected patients with early AD. However, their eligibility requirements, safety concerns, monitoring burden, and limited applicability to the broader population leave a substantial need for complementary preventive and supportive approaches [13,14].

The identification and modification of dementia risk factors therefore remain important components of prevention and clinical management [14]. In addition to physical activity [15] and cognitive stimulation [16], dietary interventions and nutritional supplementation [17] have attracted interest as possible approaches to support cognitive health. Nutraceuticals are products derived from food sources that may provide physiological benefits beyond their basic nutritional value.

Certain nutraceutical compounds have been investigated for their potential ability to influence inflammation, oxidative stress, and other biological pathways involved in brain aging. Nevertheless, evidence of biological activity should not be considered equivalent to evidence of dementia prevention or clinical efficacy.

Among the compounds currently under investigation, palmitoylethanolamide (PEA) and luteolin (Lut) have emerged as biologically plausible candidates [18]. PEA is a naturally occurring fatty acid amide with anti-inflammatory and neuroprotective properties. Lut is a flavonoid found in several fruits and vegetables that exerts antioxidant and anti-inflammatory effects [19]. Both compounds have demonstrated potentially relevant biological activities in experimental studies, although clinical evidence remains limited.

This narrative review examines the biological rationale and current experimental and clinical evidence supporting the potential role of PEA, Lut, and their co-ultramicronized formulation (PEA-Lut) in brain aging and cognitive decline. Particular attention is given to their effects on neuroinflammation, oxidative stress, mitochondrial dysfunction, cellular senescence, glial activation, and neurotrophic signaling. The innovative aspect of this review is the integration of these interconnected processes of brain aging into a comprehensive framework, highlighting the potential of PEA-Lut to act across multiple mechanisms implicated in age-related cognitive decline. Evidence derived from cellular models, animal studies, acute neurological conditions, and neurodegenerative disorders is critically discussed, with emphasis on its relevance and limitations in relation to MCI and AD. The integration of mechanistic and clinical evidence further provides insight into the translational potential of PEA-Lut and the current gaps that should be addressed in future research. Rather than establishing a preventive or therapeutic effect, this review aims to determine whether the available evidence provides a sufficient rationale for future randomized clinical trials in individuals at risk of dementia or in the early stages of cognitive impairment.

## 2. Methods

A structured literature search was conducted in PubMed/MEDLINE and the Cochrane Library. The search was based on combinations of free-text keywords using the Boolean operators AND and OR. The following terms were considered: “aging,” “dementia,” “palmitoylethanolamide,” “PEA,” “luteolin,” “co-ultramicronized palmitoylethanolamide,” “mild cognitive impairment,” “Alzheimer’s disease,” “neuroinflammation,” “Parkinson’s Disease,” “Frontotemporal Dementia”, and “traumatic brain injury”.

The search was restricted to English-language publications published from 2014 to 31 May 2026. The last search was conducted in July 2026. The search was designed to identify relevant experimental and clinical evidence, including in vitro studies, animal models, observational studies, and clinical trials. Priority was given to publications directly examining the biological effects of PEA, Lut, or PEA-Lut and their potential relevance to brain aging, cognitive impairment, and neurodegenerative disorders. Studies performed in other inflammatory or neurological conditions were considered when they provided mechanistic or translational information relevant to neuroinflammation, oxidative stress, or cognition. Additional publications were identified by screening the reference lists of relevant articles.

The first targeted search used the following Boolean strategy: (“palmitoylethanolamide” OR “PEA” OR “PEALut” OR “PEA-Lut”) AND (“dementia” OR “Alzheimer disease” OR “Parkinson disease” OR “neuroinflammation” OR “frontotemporal dementia” OR “traumatic brain injury”), identifying 190 records, of which 9 were considered sufficiently relevant for inclusion. A second targeted search used the following strategy: (“Alzheimer’s disease” OR “Alzheimer’s disease model” OR “preclinical”) AND (“palmitoylethanolamide” OR “co-ultramicronized”), identifying 98 records, of which 3 were selected for inclusion. Studies were considered eligible if they were clinical, preclinical/animal, experimental, or randomized studies addressing PEA, luteolin, or PEA-Lut in relation to neuroinflammation, aging, cognitive impairment, or neurodegenerative diseases. Editorials, commentaries, letters, reviews, and meta-analyses were excluded, as were studies that were not relevant to the objectives of the review or did not provide original experimental or clinical data. The identification and selection of relevant publications, including screening and data extraction, were independently performed by two reviewers, with discrepancies discussed and resolved by consensus. Duplicate records were identified by comparing authors, titles, publication years, and DOI and were manually removed before screening. The final selection was based on the relevance of the evidence to the objectives of the review and its scientific and translational contribution.

Given the narrative nature of this review, study selection and evidence synthesis did not follow a formal systematic-review protocol. The review first describes the principal hallmarks of aging that may contribute to cognitive decline and AD. It then examines the relationship between nutrition, nutraceuticals, and brain aging, followed by an overview of ALIAmides, PEA, and luteolin. Finally, the available preclinical and clinical evidence regarding the potential effects of PEA-Lut on chronic inflammation, neuroinflammation, and cognitive outcomes is critically discussed.

## 3. Cognitive Decline and Hallmarks of Aging

Aging is characterized by progressive molecular and cellular alterations that reduce physiological resilience and increase susceptibility to chronic disease. The original hallmarks of aging included genomic instability, telomere attrition, epigenetic alterations, loss of proteostasis, deregulated nutrient sensing, mitochondrial dysfunction, cellular senescence, stem-cell exhaustion, and altered intercellular communication [20]. More recently, impaired autophagy, chronic inflammation, and dysbiosis have also been incorporated into this conceptual framework [21]. Cognitive decline shares several biological features with the aging process. Among these, the present review focuses primarily on cellular senescence, mitochondrial dysfunction, oxidative stress, and chronic inflammation, as these interconnected processes have been implicated in brain aging, cognitive decline, and AD pathophysiology and are being investigated as potential targets for preventive and therapeutic interventions. Collectively, they may contribute to a chronic neurotoxic environment that promotes synaptic dysfunction, neuronal injury, and cognitive decline [22,23]. Experimental and clinical research has also suggested that some of these mechanisms may be amenable to pharmacological or nutritional modulation, providing a rationale for their consideration in the context of brain aging and cognitive impairment [24].

### 3.1. Cellular Senescence

Cellular senescence is a state of stable cell-cycle arrest in which cells lose their ability to proliferate while remaining metabolically active. Although senescence has important physiological functions, including tissue repair and tumor suppression, the accumulation of senescent cells during aging may contribute to tissue dysfunction and age-related diseases, including cognitive decline and AD [25,26]. Senescence is not limited to the loss of replicative capacity but involves complex changes in cellular metabolism, gene expression, morphology, and epigenetic regulation [27]. Senescent cells commonly become flatter and larger [28] and may exhibit increased senescence-associated β-galactosidase activity [29], increased expression of the cyclin-dependent kinase inhibitor p16 [30], and the formation of senescence-associated heterochromatin foci [31]. Mitochondrial dysfunction is also a characteristic feature of cellular senescence and may be associated with these epigenetic alterations [32]. In addition, senescent cells may acquire a senescence-associated secretory phenotype (SASP), characterized by the release of pro-inflammatory cytokines, growth factors, chemokines, and proteases [33].

Evidence suggests that several brain cell populations, including astrocytes, microglia, endothelial cells, oligodendrocyte progenitor cells, and possibly neurons, may acquire senescent or senescence-like features during aging and neurodegenerative disease [34].

Although the overall number of astrocytes in the human brain may not substantially change with aging [35], these cells may accumulate lipofuscin [36] and develop SASP-related alterations [37]. Oxidative metabolism in astrocytes also appears to increase with age, potentially reducing their ability to provide neurons with metabolic support [38]. Consistently, astrocytes exposed to oxidative stress in vitro exhibit several features of cellular senescence [39]. Microglia also undergo age-related structural and functional changes, including cytoplasmic abnormalities and dystrophic morphology [40]. Following repeated exposure to stressful stimuli, such as lipopolysaccharide, cultured microglial cells may develop a senescence-like phenotype characterized by increased β-galactosidase activity [41] and the formation of senescence-associated heterochromatin foci [42].

Neurons are terminally differentiated cells and therefore do not undergo classical replicative senescence [43]. Nevertheless, experimental evidence indicates that post-mitotic neurons may develop a senescence-like phenotype [44,45].

Under oxidative and metabolic stress, neurons may accumulate lipofuscin, misfolded proteins, and other cellular waste products. Mitochondrial dysfunction and oxidative stress have also induced senescence-like features in neuronal cell models, including PC12 and SH-SY5Y cells [46]. Evidence of cellular senescence has also been reported in human AD brain tissue. Increased DNA damage and senescence-associated markers have been observed in astrocytes from brain regions vulnerable to AD [47]. Expression of p16 and SASP-related factors, including matrix metalloproteinase 3, has been reported to be higher in cortical tissue from individuals with AD than in age-matched controls. Microglia from AD brains may similarly exhibit increased senescence-associated markers, shorter telomeres, and a dystrophic phenotype that may precede the development of tau pathology [13]. Telomere shortening has been proposed as a marker of biological aging, although its relationship with AD remains uncertain. Some studies have reported shorter telomeres in peripheral blood mononuclear cells from individuals with AD than in age-matched controls. T-cell telomere length has also been positively associated with cognitive performance and inversely associated with circulating tumor necrosis factor-α, suggesting a possible relationship among telomere attrition, immune dysfunction, and cognitive decline [48]. Overall, cellular senescence may contribute to brain aging through the accumulation of dysfunctional cells, SASP-mediated inflammation, impaired cellular support, and reduced tissue resilience [49,50].

### 3.2. Mitochondrial Dysfunction and Oxidative Stress

Mitochondria are essential for cellular function. In addition to producing energy through oxidative phosphorylation, they contribute to phospholipid and heme synthesis, calcium homeostasis, cellular signaling, apoptosis, and other forms of regulated cell death [51].

Mitochondrial dysfunction is considered an important feature of biological aging and has been implicated in several age-related conditions, including metabolic, cardiovascular, neoplastic, and neurodegenerative diseases.

During aging, mitochondria may accumulate mitochondrial DNA mutations and develop impaired respiratory function. These alterations can increase the production of reactive oxygen species (ROS) and reduce cellular energy availability, potentially causing oxidative damage to lipids, proteins, and nucleic acids [52]. Oxidative stress occurs when the production of reactive species exceeds the capacity of cellular antioxidant and repair systems. It has been implicated in brain aging and in the pathophysiology of several neurodegenerative disorders, including AD.

The brain is particularly vulnerable to oxidative injury because of its high oxygen consumption, substantial lipid content, abundance of polyunsaturated fatty acids, and relatively limited antioxidant reserves. Increased ROS production, mitochondrial dysfunction, altered metal homeostasis, and reduced antioxidant defenses may impair synaptic activity and neurotransmission, thereby contributing to cognitive dysfunction [53]. Oxidative stress and neuroinflammation are closely interconnected. Activated microglia produce inflammatory and reactive mediators, including nitric oxide (NO) and superoxide. These molecules may react to form peroxynitrite, a highly reactive compound capable of inducing DNA damage, lipid peroxidation, protein modification, mitochondrial dysfunction, and neuronal injury. Reactive astrocytes may also release NO and other inflammatory mediators into the extracellular environment, further contributing to oxidative damage and impaired neuronal survival [54]. Increased concentrations of oxidative damage markers, including advanced oxidation protein products, have been reported in individuals with MCI and AD.

The high lipid and iron content of brain tissue may further increase its susceptibility to free-radical-mediated injury [55]. However, many oxidative biomarkers are nonspecific, and their circulating concentrations may not directly reflect oxidative processes within the central nervous system [55]. Glutathione (GSH) is one of the principal endogenous antioxidant systems and plays an essential role in maintaining cellular redox homeostasis in the brain. Redox balance also influences the function of redox-sensitive proteins, including N-methyl-D-aspartate receptors (NMDARs). These glutamate receptors are involved in hippocampal synaptic plasticity and are essential for long-term potentiation, learning, and memory [56]. Overall, mitochondrial dysfunction and oxidative stress appear to contribute to brain aging and AD through interconnected effects on energy metabolism, cellular signaling, neuroinflammation, synaptic function, and neuronal survival [57]. These mechanisms provide a biological rationale for investigating interventions capable of modulating oxidative and mitochondrial pathways. Nevertheless, evidence that changes in oxidative biomarkers translate into clinically meaningful cognitive benefits remains limited.

### 3.3. Inflammation

Inflammation is a coordinated biological response to infection, tissue damage, toxic compounds, and other harmful stimuli. Its principal functions are to eliminate or contain the source of injury and initiate tissue repair [58]. Although an appropriately regulated inflammatory response is protective, its persistence or inadequate resolution may result in chronic inflammation and contribute to tissue dysfunction.

Aging is associated with persistent, low-grade systemic inflammation, commonly referred to as inflammaging. This condition is characterized by a moderate increase in circulating pro-inflammatory mediators and is thought to result from several age-related processes, including cellular senescence, mitochondrial dysfunction, impaired autophagy, immune dysregulation, and the accumulation of cellular damage. In the context of aging and AD, an imbalance between pro-inflammatory and inflammation-resolving pathways may promote a chronic neuroinflammatory environment with glial activation and sustained release of inflammatory mediators [59].

Neuroinflammation is increasingly recognized as an important component of AD pathophysiology. Reactive microglia and astrocytes are commonly found in proximity to amyloid-β deposits and other areas of neuronal injury. Changes in inflammatory signaling may occur before the onset of overt cognitive symptoms, although their timing, magnitude, and contribution to disease progression remain incompletely understood [60,61]. Persistent microglial activation may impair amyloid-β clearance, promote synaptic dysfunction, and amplify local inflammatory responses. Conversely, amyloid-β accumulation and neuronal injury may further activate microglia, establishing a self-perpetuating cycle of inflammation and neurodegeneration [61].

Circulating inflammatory mediators have been investigated as possible biomarkers of aging and cognitive decline [62]. Compared with younger adults, older individuals may exhibit higher circulating concentrations of interleukin (IL)-1β, IL-6, tumor necrosis factor-α (TNF-α), and C-reactive protein, together with alterations in anti-inflammatory mediators such as IL-10 and transforming growth factor-β [63]. However, these markers are nonspecific and may be influenced by comorbidities, adiposity, infections, medication use, and other age-related factors. Their circulating levels should therefore not be considered direct measures of neuroinflammation.

Aging is also associated with changes in the morphology, distribution, and function of glial cells [64]. Astrocytes, oligodendrocytes, and microglia contribute to neuronal activity, metabolic support, myelination, immune surveillance, and maintenance of central nervous system homeostasis [65]. When persistently activated or dysfunctional, glial cells may release cytokines, chemokines, reactive oxygen and nitrogen species, eicosanoids, excitatory mediators, and proteases that can damage neurons and oligodendrocytes [66]. This response may sustain and amplify local neuroinflammation.

Astrocytes represent a major component of the glial compartment and participate in amino acid and lipid metabolism, ion and water homeostasis, antioxidant defense, neurotransmitter recycling, and regulation of inflammatory responses. Disruption of these functions may alter neuronal activity and increase susceptibility to neurological disease [67]. In AD, both microglia and astrocytes interact with amyloid-β. Glial dysfunction may impair amyloid-β clearance, while amyloid accumulation may further stimulate the production of inflammatory mediators and contribute to neurodegeneration [68]. Overall, chronic systemic inflammation and neuroinflammation appear to interact with oxidative stress, mitochondrial dysfunction, and cellular senescence during brain aging and AD. Although inflammatory pathways represent biologically plausible therapeutic targets, their effects may vary according to disease stage, cell type, and local microenvironment. These considerations support the investigation of compounds capable of modulating, rather than completely suppressing, inflammatory responses.

Cellular senescence, oxidative stress, and inflammation are closely interconnected and may reinforce one another. Oxidative stress promotes the generation and accumulation of ROS, which may induce cellular senescence [69]. Inflammatory processes may also increase oxidative stress, which can, in turn, amplify inflammatory signaling through several pathways [70]. Understanding the interactions among these mechanisms may contribute to the identification of new preventive and therapeutic strategies for AD. Given their involvement in brain aging and cognitive decline, interventions capable of modulating inflammation, oxidative balance, and cellular resilience warrant further investigation. Within this framework, nutrition represents a potentially modifiable factor that may influence several of these interconnected biological pathways.

**Figure 1:**
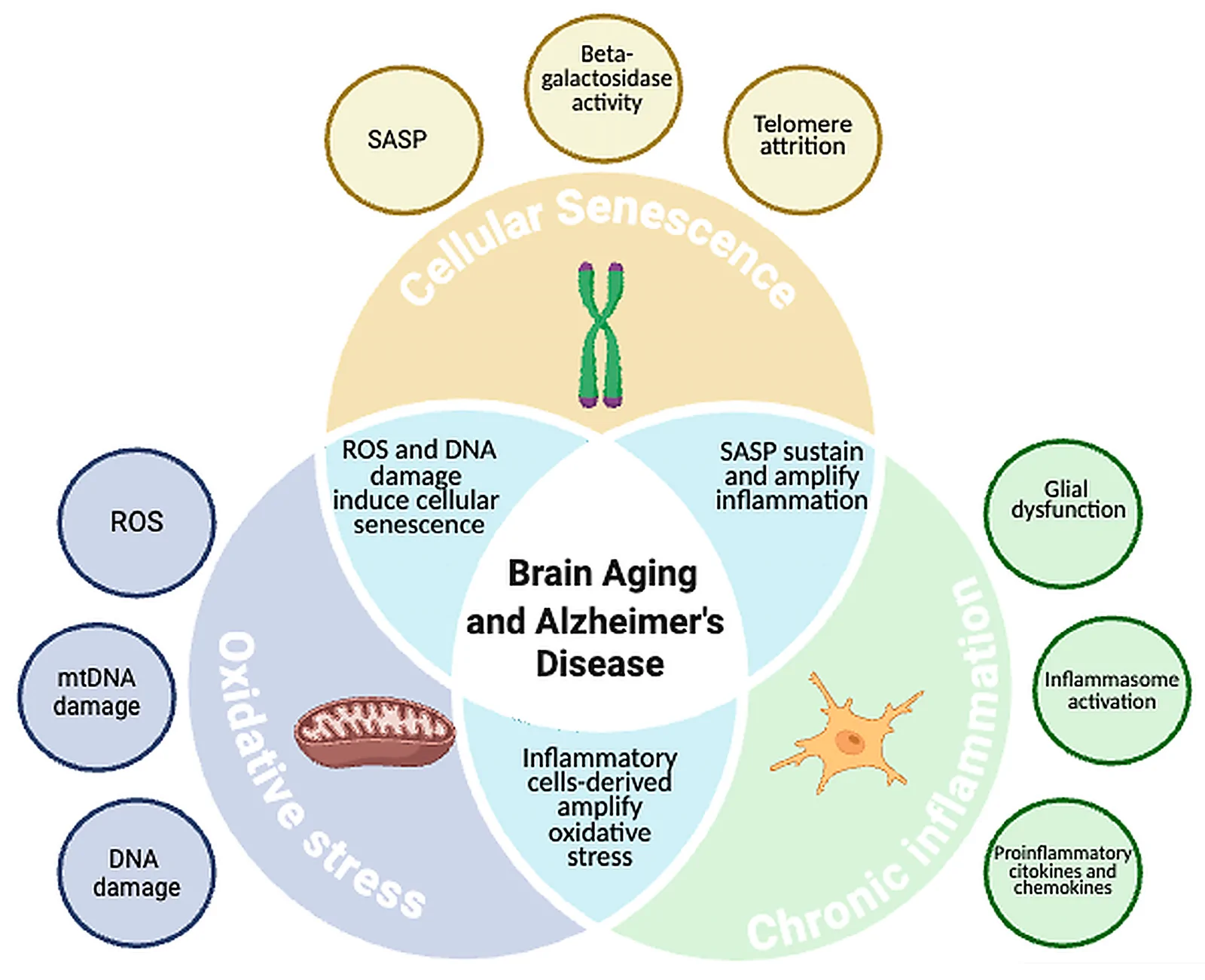
Interconnected hallmarks of brain aging: cellular senescence, chronic inflammation, and oxidative stress.

The interaction among the principal hallmarks of aging potentially involved in brain aging is summarized in Figure 1.

The conceptual diagram illustrates three potentially interconnected hallmarks of brain aging: cellular senescence, chronic inflammation, and oxidative stress. These processes are tightly linked, forming a dynamic positive feedback loop that perpetuates and accelerates brain aging. Targeting even one of these mechanisms may modulate the others, ultimately influencing the overall trajectory of brain aging.

## 4. Alzheimer’s Disease: From Nutrition to Nutraceuticals

The global prevalence of dementia is increasing as the population ages, with AD remaining its most common cause. Although recently introduced disease-modifying therapies may modestly slow cognitive and functional decline in selected patients with early AD, no treatment has been shown to completely prevent the disease. Prevention strategies therefore continue to focus on potentially modifiable risk factors and on the biological mechanisms that contribute to cognitive decline [71]. Diet may influence cognitive health through its effects on cardiometabolic risk, insulin sensitivity, energy metabolism, inflammation, and oxidative stress. Excess adiposity and dietary patterns characterized by high consumption of refined carbohydrates, saturated fats, and highly processed foods have been associated with an increased risk of cognitive decline and dementia [72,73].

However, these associations are influenced by several demographic, clinical, behavioral, and socioeconomic factors, and do not establish a direct causal relationship. Caloric restriction and related metabolic interventions have been associated with increased lifespan and reduced age-related pathology in several experimental models [74]. Nevertheless, their effects on the prevention of AD in humans remain uncertain, and excessive dietary restriction may be harmful in older adults at risk of malnutrition, sarcopenia, or frailty. Dietary composition also influences metabolic homeostasis. High intakes of refined sugars and saturated fats may adversely affect insulin sensitivity [75], whereas dietary patterns with a different macronutrient composition may alter the relative use of glucose and fatty acids as energy substrates [76].

The clinical relevance of these metabolic changes to dementia prevention has not yet been fully established. Nutritional requirements may change with age, and deficiencies in certain nutrients can adversely affect cognition and neurological function [77]. Dietary patterns rich in fruit, vegetables, legumes, nuts, whole grains, and fish have been associated with better cognitive outcomes and a lower risk of dementia [78,79]. These foods provide polyphenols, unsaturated fatty acids, vitamins, minerals, and other compounds with potential antioxidant and anti-inflammatory properties [80,81]. Adequate concentrations of vitamins, such as vitamin B12 and vitamin E, as well as minerals, including zinc and magnesium, contribute to normal neurological function [82,83], but supplementation in individuals without a documented deficiency has not consistently been shown to prevent cognitive decline. Conversely, dietary patterns high in saturated fats, refined sugars, and highly processed foods have been associated with poorer cognitive outcomes [84].

Research examining the relationship among nutrition, aging, and AD may help identify dietary strategies capable of supporting cognitive health and reducing the burden of modifiable risk factors [85]. Nutritional interventions may also influence cellular senescence, inflammation, oxidative stress, and metabolic dysfunction, although the magnitude and clinical relevance of these effects remain uncertain [86]. In this context, nutraceuticals have attracted increasing scientific interest. A nutraceutical has been broadly defined as “a food or part of a food that provides health benefits in addition to its nutritional content” [87].

The term combines the concepts of nutrition and pharmaceuticals and generally refers to food-derived compounds used with the aim of producing physiological effects beyond basic nutrition. Nutraceuticals have been investigated for potential anti-inflammatory, antioxidant, metabolic, prebiotic, and other biological effects [88].

Nevertheless, the composition, bioavailability, regulatory oversight, and level of supporting evidence vary considerably among products. In the context of brain aging and AD, compounds such as omega-3 fatty acids, vitamins, minerals, carotenoids, and polyphenols have been studied for their possible effects on cognitive function, inflammation, oxidative damage, and neurodegeneration [89]. Although some findings are encouraging, evidence of biological activity or epidemiological association should not be interpreted as proof of clinical efficacy or dementia prevention. Among the dietary patterns investigated in relation to cognitive health, the Mediterranean diet has received particular attention because of its high content of plant-derived bioactive compounds and unsaturated fatty acids [90]. It is characterized by a high intake of fruit, vegetables, legumes, nuts, minimally processed cereals, and olive oil; moderate consumption of fish and dairy products; and limited consumption of red and processed meat [91].

Observational studies have associated greater adherence to this dietary pattern with more favorable cognitive outcomes, although residual confounding and differences in dietary assessment limit causal interpretation.

The potential benefits of the Mediterranean diet are unlikely to depend on a single nutrient. Rather, they may reflect the combined and potentially synergistic effects of fiber, polyphenols, unsaturated fatty acids, vitamins, minerals, and other bioactive compounds [90]. These components may influence vascular and metabolic health, oxidative balance, inflammatory signaling, and gut microbiota, all of which have been implicated in brain aging.

Nutritional compounds have also been investigated for their ability to modulate cellular processes involved in neurogenesis, synaptic plasticity, neurotransmission, neuroinflammation, oxidative stress, and neuronal survival [92]. Nutraceuticals, including omega-3 fatty acids [93], B vitamins [94], polyphenols [95], carotenoids [96], and antioxidant compounds [97], have therefore been examined in relation to cognitive decline and dementia.

Among these compounds, PEA represents a biologically plausible candidate for further investigation. PEA is an endogenous lipid mediator synthesized in response to cellular stress and tissue injury. It contributes to the regulation of immune and inflammatory responses and may promote the resolution of inflammation [98]. PEA can also be administered exogenously, either alone or in combination with antioxidant compounds such as luteolin, with the aim of providing complementary biological effects [99].

The following section examines the biological characteristics of PEA and the rationale for its co-administration with Lut.

### 4.1. The ALIAmides and Palmitoylethanolamide

The term ALIAmides derives from “Autacoid Local Injury Antagonist amides” and was introduced by Rita Levi-Montalcini and colleagues to describe a group of endogenous bioactive N-acylethanolamines involved in the local regulation of inflammatory and tissue responses. Among these compounds, PEA has been extensively investigated for its anti-inflammatory, analgesic, and neuroprotective properties [99]. PEA is an endogenous N-acylethanolamine and bioactive lipid mediator synthesized in response to cellular stress or tissue injury. It is thought to contribute to the maintenance and restoration of tissue homeostasis by regulating immune-cell activation and inflammatory signaling. Its reported biological activities include anti-inflammatory, analgesic, immunomodulatory, anticonvulsant, and neuroprotective effects [100]. However, the clinical relevance of these effects varies according to the condition studied and the formulation administered.

PEA does not act as a direct antioxidant or free-radical scavenger. Its antioxidant effects, when observed, are therefore likely to be indirect and related to modulation of inflammatory pathways, cellular stress responses, and endogenous defense mechanisms. Native, non-micronized PEA also has limited aqueous solubility and variable gastrointestinal absorption, partly because of its lipophilic structure and heterogeneous particle size [99]. Micronized and ultramicronized formulations have been developed to reduce particle size and improve dissolution, dispersion, and potentially oral bioavailability. Experimental studies have reported greater biological activity of micronized or ultramicronized PEA than non-micronized PEA in some models of inflammatory and neuropathic pain [101,102].

Nevertheless, evidence directly comparing the pharmacokinetic and clinical performance of different formulations remains limited.

Co-ultramicronization of PEA with Lut may modify the physicochemical properties of the combined formulation. It has been proposed that interactions between the two molecules, including hydrogen bonding, may reduce PEA crystallization and improve particle stability and dispersion. PEA and Lut also have potentially complementary biological properties, providing a rationale for their combined administration [101]. Evidence that the co-ultramicronized formulation is clinically superior to either compound administered alone remains insufficient.

Lut (3′,4′,5,7-tetrahydroxyflavone) is a naturally occurring flavone found in several plant-derived foods, including celery, peppers, broccoli, and thyme. Experimental studies have reported antioxidant, anti-inflammatory, analgesic, and neuroprotective activities in both in vitro and in vivo models [99,103,104].

Lut may modulate the production of pro-inflammatory cytokines and nitric oxide, activate endogenous antioxidant pathways, and inhibit nuclear factor-κB signaling [105]. These findings provide a mechanistic rationale for its combination with PEA, although their translation into clinically meaningful cognitive benefits has not yet been established.

### 4.2. Pharmacokinetics of Palmitoylethanolamide and Luteolin

Pharmacokinetic data on PEA remain limited. In particular, its oral bioavailability, apparent volume of distribution, systemic clearance, and routes of elimination have not yet been clearly established. Following oral administration, PEA is likely to undergo rapid enzymatic hydrolysis, primarily mediated by fatty acid amide hydrolase and N-acylethanolamine acid amidase, although the extent and clinical relevance of these processes in humans remain incompletely characterized [106,107]. In healthy volunteers, administration of 300 mg micronized PEA produced an approximately twofold increase in plasma PEA concentrations at 2 h, with levels returning toward baseline over subsequent hours. These findings indicate transient systemic exposure but do not allow determination of absolute oral bioavailability [107].

Preclinical studies indicate that orally administered PEA can reach peripheral tissues and, in small amounts, the central nervous system. In rats, radiolabeled PEA was detected in the brain shortly after administration and showed a heterogeneous regional distribution, with preferential accumulation in the hypothalamus. Radioactivity was also detected in the pituitary and adrenal glands, reflecting distribution beyond the brain parenchyma [107,108]. In a subsequent study in rats using ^13^C-labeled PEA, orally administered ultramicronized PEA produced higher plasma concentrations than the non-micronized formulation and resulted in measurable increases in PEA levels in the brain and spinal cord [106]. However, these findings were obtained in rodents and should not be interpreted as definitive evidence of clinically relevant central nervous system exposure in humans.

The poor aqueous solubility of PEA is considered an important determinant of its dissolution-rate-limited oral absorption. Accordingly, micronization, ultramicronization, and other formulation strategies may enhance dissolution and systemic exposure. Consistent with this rationale, a recent comparative study in rats reported increased Cmax and substantially greater overall exposure for micronized and water-dispersible PEA than for non-micronized PEA; the water-dispersible formulation produced an AUC more than 16-fold higher than that of the non-micronized preparation [109]. These findings remain formulation-specific and preclinical, and complete absolute pharmacokinetic values are required for a direct comparison of Cmax, AUC, and Tmax across preparations.

Overall, current evidence suggests that the pharmacokinetic profile of orally administered PEA is strongly formulation-dependent, although the magnitude and clinical relevance of these differences remain incompletely defined. Direct pharmacokinetic comparisons among non-micronized, micronized, ultramicronized, and water-dispersible preparations in humans are still limited. Moreover, available formulation data should not be directly extrapolated to PEA-Lut, for which dedicated pharmacokinetic studies are required.

### 4.3. Safety and Tolerability

The available clinical evidence on the safety of PEA and PEA-Lut is limited and heterogeneous. Most randomized controlled trials and observational studies have not reported detailed safety data in a standardized manner, and information on adverse events, treatment discontinuations, and reasons for discontinuation is often incomplete.

In a randomized, single-center, quadruple-blinded, placebo-controlled trial with 70 participants with diabetic peripheral neuropathic pain, 600 mg/day of PEA was administered for 8 weeks. The study reported no clinically relevant changes in independently assessed laboratory markers of electrolytes, kidney function, or liver function. Fasting glucose levels increased significantly over 8 weeks in both groups, and hematology parameters remained stable except for a small but significant difference in eosinophil count. Several mild, short-duration adverse events were reported, including intermittent mild headache (*n* = 1 per group), constipation (*n* = 1, active), urticaria for 5 days (*n* = 1, active), a severe fatigue episode (*n* = 1, placebo), and respiratory infections not requiring additional medications (*n* = 3, placebo). All adverse events resolved during the study period, and no participant was withdrawn because of an adverse event [110].

In a double-blind, randomized, placebo-controlled study, 80 otherwise healthy adults with recurrent migraine attacks received 600 mg of PEA or a placebo at the onset of migraine symptoms. Although the study was primarily designed to assess migraine outcomes, safety and gastrointestinal tolerability were also monitored. No adverse events were reported in either group during the study, and no clinically relevant tolerability concerns were identified. However, these findings should be interpreted in the context of the relatively healthy study population, the short observation period for each treatment episode, and the maximum follow-up of approximately 4 months [111].

In a monocentric, randomized, double-blind, placebo-controlled phase II trial, 48 patients with probable frontotemporal dementia were randomized to receive co-ultramicronized PEA-Lut, containing 700 mg of PEA and 70 mg of Lut twice daily, or placebo for 24 weeks. Twenty-five patients were assigned to the PEA-Lut group, and 23 to the placebo group; 45 patients (93%) completed the study. Treatment adherence was high in both groups, reaching 91.4% in the PEA-Lut group and 90.6% in the placebo group. Adverse events were generally similar between groups. In the PEA-Lut group, headache and nausea were each reported in two patients (8%), dizziness in one patient (4%), severe urinary tract infection in one patient (4%), and severe COVID-19 infection with pneumonia in one patient (4%). In the placebo group, headache was reported in three patients (13.0%), nausea in one patient (4.3%), diarrhea in one patient (4.3%), and severe COVID-19 infection in one patient (4.3%). Treatment discontinuation because of adverse events occurred in two patients receiving PEA-Lut (8%) and one patient receiving placebo (4.3%). No deaths, treatment-related serious adverse events, or cases of unblinding were reported. Although these findings indicate generally comparable short-term tolerability between groups, they should be interpreted cautiously given the small sample size, monocentric design, and limited 24-week follow-up [112].

In a multicenter, double-blind, randomized, placebo-controlled trial, 185 individuals with persistent post–COVID-19 olfactory dysfunction were assigned to receive oral ultramicronized PEA-Lut (770 mg/day) combined with olfactory training (*n* = 130) or placebo plus olfactory training (*n* = 55) for 90 days. The study primarily evaluated olfactory outcomes and reported greater improvement in the intervention group than in the placebo group, with no major treatment-related safety signals, and adverse events were comparable between the PEA-Lut and placebo groups [113].

In a prospective, randomized, open-label, controlled clinical trial, 60 patients with acute ischemic stroke undergoing thrombolysis were assigned to receive either standard thrombolytic therapy alone or standard therapy plus oral co-ultramicronized PEA-Lut (700 mg of PEA plus 70 mg of luteolin twice daily) for 90 days. No PEA-Lut-related adverse events were observed during the study, and routine blood chemistry and hematological analyses performed at baseline and after 90 days revealed no treatment-related deviations from the normal range. Although these findings support the short-term tolerability of PEA-Lut when administered in addition to thrombolytic therapy, they should be interpreted cautiously because of the small sample size, open-label, single-center design, short follow-up, and limited power to detect rare or delayed adverse events [114].

Overall, the available clinical evidence has not identified major treatment-related safety signals with PEA or co-ultramicronized PEA-Lut during the limited treatment and observation periods evaluated. This interpretation is consistent with a recent PRISMA 2020–compliant systematic review of 47 randomized controlled trials and 48 eligible study outputs across a broad range of clinical conditions, which found that PEA—particularly in micronized, ultramicronized, and cold-water-dispersible formulations—was generally well tolerated and not associated with a consistent excess of adverse events compared with placebo or control treatments [115]. Similarly, a systematic review and meta-analysis restricted to 11 double-blind randomized controlled trials of PEA for chronic pain, including 774 patients, found no major adverse effects attributable to PEA. In the narrative synthesis of safety outcomes, six studies reported no treatment-associated adverse events, three found no differences in adverse-event profiles between PEA and comparator groups, and one reported only a very low incidence of mild, transient gastrointestinal symptoms [116].

However, the underlying evidence is heterogeneous with respect to clinical populations, formulations, doses, treatment duration, and the ascertainment and reporting of adverse events. Although adverse events, treatment discontinuations, and laboratory safety parameters were described in some studies, these outcomes were not assessed or reported uniformly. Consequently, the current data are insufficient to exclude rare, delayed, or formulation-specific adverse effects; to establish the long-term safety profile of PEA or PEA-Lut; or to define contraindications and potential drug–drug interactions, particularly in older adults with multimorbidity and polypharmacy. Moreover, clinical safety data on Lut administered alone remain scarce. Larger, adequately powered, preferably multicenter trials in populations representative of those likely to receive PEA-Lut in clinical practice are therefore needed. These studies should include longer treatment and follow-up periods; standardized collection and reporting of adverse events; documentation of treatment discontinuations and their causes; laboratory safety assessments; and prospective evaluation of clinically relevant drug–drug interactions.

## 5. PEA and Luteolin, Chronic Inflammation and Cognitive Impairment: What Evidence?

A growing body of preclinical and clinical evidence suggests that PEA-Lut may exert neuroprotective effects by modulating chronic inflammation, oxidative stress, and glial activation [105]. Given the involvement of these interconnected mechanisms in the pathophysiology of several neurological disorders, PEA-Lut has been studied in a wide range of conditions characterized by neuroinflammation and cognitive impairment and, in particular, for its potential ability to modulate neuroinflammatory processes [117] across neurological conditions, including traumatic brain injury (TBI), stroke, Parkinson’s disease (PD), frontotemporal dementia, and AD [104,118].

Evidence from these conditions may provide relevant mechanistic and safety information regarding the effects of PEA-Lut on neuroinflammation and neuronal homeostasis; however, such findings should not be interpreted as evidence of established clinical efficacy or long-term safety in cognitive impairment or dementia.

The following sections summarize the available evidence regarding the potential therapeutic effects of PEA-Lut in cerebrovascular and major neurodegenerative diseases.

### 5.1. PEA-Lut and Traumatic Brain Injury

PEA-Lut has been investigated in experimental models of TBI, a condition in which secondary neuroinflammatory and oxidative processes contribute to the progression of tissue damage. Although TBI differs from age-related neurodegenerative disease, these models may provide information on the effects of PEA-Lut on neuroinflammation, neuronal survival, and cognitive recovery.

Cordaro et al. evaluated PEA-Lut in a mouse model of controlled cortical impact. Treatment was associated with improved motor and cognitive performance, reduced lesion volume, and modulation of apoptosis-related pathways, cytokine release, reactive oxygen species, chymase and tryptase activity, nitrotyrosine formation, and autophagy-associated signaling [119]. These findings suggest that PEA-Lut may modulate several components of the secondary injury response, although the relative contribution of each mechanism remains uncertain. Potential neuroregenerative effects of PEA-Lut were subsequently investigated in an experimental TBI model, together with a preliminary clinical evaluation in patients with moderate TBI. In mice, PEA-Lut increased neurogenesis, restored markers of immature and mature neurons, and upregulated neurotrophic factors, with associated improvements in memory performance [120]. However, the small sample sizes and the potential confounding effects related to hospitalization should be considered when interpreting these findings.

Overall, TBI models suggest that PEA-Lut may influence neuroinflammation, oxidative injury, neurotrophic signaling, and cognitive recovery after acute brain injury. Nevertheless, given the substantial differences between TBI and AD, these findings should be considered supportive mechanistic evidence rather than direct evidence for the treatment or prevention of age-related cognitive impairment.

### 5.2. PEA-Lut and Stroke

The effects of PEA-Lut have also been investigated in preclinical models and clinical populations with ischemic stroke, in which neuroinflammation, oxidative stress, and secondary neuronal injury may influence neurological and cognitive recovery.

Caltagirone et al. evaluated PEA-Lut in an experimental model of cerebral ischemia and a clinical cohort of patients undergoing post-stroke rehabilitation. In a rat model of middle cerebral artery occlusion, treatment reduced cerebral edema and infarct volume, improved neurological performance, and decreased selected inflammatory and astrocytic markers. In the clinical component, 250 patients undergoing inpatient or outpatient neurorehabilitation received co-ultramicronized PEA-Lut for 60 days, with improvements in neurological status, cognitive performance, spasticity, pain, and independence in activities of daily living [121]. However, the observational design and absence of a randomized placebo-controlled comparison limit causal interpretation, as improvements may have been influenced by rehabilitation, spontaneous recovery, stroke severity, concomitant treatments, and other clinical factors. The findings should therefore be considered preliminary evidence of feasibility and possible clinical benefit rather than proof of efficacy.

A subsequent clinical study evaluated PEA-Lut as an adjunct to thrombolytic treatment in acute ischemic stroke. Improvements over time were observed in neurological status, independence in activities of daily living, disability, and cognitive performance assessed using the Mini-Mental State Examination and Montreal Cognitive Assessment, with some outcomes appearing more favorable among patients receiving PEA-Lut than in those receiving standard treatment alone [114]. However, interpretation requires consideration of the study design, sample size, allocation method, baseline comparability, and follow-up duration. Overall, available stroke studies suggest that PEA-Lut may have adjunctive effects on neurological and cognitive recovery. However, post-stroke cognitive impairment differs from MCI due to AD in its pathophysiology and clinical trajectory, and improvements during stroke rehabilitation cannot be assumed to indicate prevention of progressive neurodegenerative cognitive decline. Controlled trials with prespecified cognitive outcomes are required to clarify the clinical relevance of these findings.

### 5.3. PEA-Lut and Parkinson’s Disease

PEA-Lut has also been investigated in models of PD, a neurodegenerative disorder in which neuroinflammation, oxidative stress, mitochondrial dysfunction, and impaired protein homeostasis may contribute to dopaminergic neuronal injury. In an experimental mouse model of PD, daily PEA-Lut administration for up to 7 days was associated with preservation of tyrosine hydroxylase immunoreactivity and with modulation of astrocyte activation, pro-inflammatory cytokines, inducible nitric oxide synthase, and autophagy-related pathways [122]. A clinical case report subsequently described the use of PEA-Lut as an adjunct to carbidopa/levodopa in a patient with PD, with reported improvements in dyskinesia and camptocormia [123].

Given the uncontrolled nature of a single-patient report, the observed changes cannot be attributed with certainty to PEA-Lut and may have been influenced by fluctuations in PD symptoms, concomitant treatment, placebo effects, or other clinical factors. Therefore, the results remain limited and cannot be generalized to the broader population.

Evidence from PD therefore provides additional mechanistic information regarding the possible effects of PEA-Lut on neuroinflammation, oxidative stress, and autophagy, but limited clinical evidence is insufficient to establish efficacy or support extrapolation to cognitive impairment or AD.

### 5.4. PEA-Lut and Frontotemporal Dementia

Assogna et al. investigated the effects of PEA-Lut in 17 patients with probable frontotemporal dementia, assessing neuropsychological, behavioral, and neurophysiological outcomes before and after 4 weeks of treatment. Changes were reported in Neuropsychiatric Inventory and Frontal Assessment Battery scores, together with changes in long-interval intracortical inhibition assessed by transcranial magnetic stimulation, providing preliminary information on possible effects on behavioral and cortical function [124].

However, the study was limited by its small sample size, short treatment duration, and absence of a randomized placebo-controlled comparison. Practice effects, fluctuations in behavioral symptoms, concomitant therapies, and treatment-related expectations may have contributed to the observed changes. The findings should therefore be regarded as preliminary and hypothesis-generating.

The same authors subsequently conducted a randomized, placebo-controlled phase II trial involving 48 patients with frontotemporal dementia. Over the 24 weeks, PEA-Lut was associated with a slower rate of clinical decline compared with placebo, with potential benefits observed in global disease severity, functional status, and language performance [112]. Although these findings are encouraging, larger and long-term controlled studies are needed to confirm their reproducibility and clinical relevance.

Although frontotemporal dementia differs from AD in its underlying pathology and clinical presentation, these studies may provide preliminary insights into the effects on cognitive, behavioral, and neurophysiological outcomes in a neurodegenerative dementia population. The small sample sizes, single-center design, and short follow-up periods limit assessment of the long-term effects of PEA-Lut. Larger controlled trials are required to determine whether the observed changes are reproducible and clinically meaningful.

### 5.5. PEA-Lut and Alzheimer’s Disease

In the context of AD, Paterniti et al. investigated the effects of PEA-Lut using in vitro and ex vivo experimental models. Treatment was associated with reduced amyloid-β-induced astrocyte activation and attenuation of cellular injury in glial cells [125]. These findings suggest that PEA-Lut may modulate glial responses to amyloid-β, although they do not establish whether comparable effects occur in the human brain or translate into cognitive benefit.

The effects of PEA-Lut administered during an AD-like prodromal phase were subsequently investigated in rats exposed to amyloid-β. In this model, amyloid-β induced astrocyte and microglial activation together with increased expression of pro-inflammatory mediators. Early PEA-Lut administration was associated with reduced astrogliosis and microgliosis, lower expression of selected pro-inflammatory cytokines and enzymes, and increased expression of neurotrophic factors, including brain-derived neurotrophic factor and glial cell line-derived neurotrophic factor [126]. These findings are consistent with a possible neuroprotective effect and suggest that the timing of administration may influence the biological response.

The same research group subsequently evaluated PEA-Lut in cultured astrocytes and oligodendrocytes exposed to amyloid-β1–42. The formulation attenuated some of the morphological and functional changes induced by amyloid-β in both cell types [127]. This study provides further preclinical evidence that the effects of PEA-Lut may extend beyond astrocytes to other glial populations involved in neuronal support and myelin homeostasis.

Then, more recently, they investigated the effects of ultramicronized PEA-Lut in a triple-transgenic mouse model of AD. Chronic treatment was associated with restoration of astrocyte-neuron metabolic homeostasis, including alterations in lactate-glutamate and taurine metabolism, as well as enhanced Klotho/FGF21 signaling, processes that have been implicated in the pathophysiology of AD [128].

These findings provide further preclinical evidence that PEA-Lut may influence metabolic pathways involved in astrocyte-neuron coupling and AD-related cellular dysfunction. However, as this study was conducted in an animal model, its findings cannot be directly extrapolated to the effects of PEA-Lut or to clinical outcomes in patients with AD.

Collectively, these experimental studies provide a biological rationale for investigating PEA-Lut in AD-related neuroinflammation and amyloid-β-induced cellular injury. Nevertheless, the available evidence is currently limited to cellular, ex vivo, and animal models. These models reproduce only selected aspects of the complex human disease. In particular, animal models are based on experimentally induced or genetically engineered pathological features that cannot fully recapitulate the multifactorial pathophysiology, progressive course, and clinical heterogeneity of human dementia. Moreover, differences in disease pathophysiology, pharmacokinetics, metabolism, and treatment exposure between experimental models and humans may further limit the direct translation of preclinical findings.

These models cannot establish whether PEA-Lut prevents cognitive decline, delays progression from MCI to dementia, or improves clinical outcomes in established AD.

Human studies specifically enrolling individuals with biomarker-confirmed MCI due to AD or AD dementia are lacking. Future clinical research should therefore focus on multicenter, randomized, placebo-controlled trials with long-term follow-up to evaluate clinically meaningful cognitive and functional outcomes, treatment duration, dose–response relationships, safety, adherence, and potential interactions with standard pharmacological therapies. Particular attention should be given to individuals with MCI, as this population may represent a relevant stage at which to investigate whether PEA-Lut could slow cognitive and clinical progression toward dementia. The inclusion of validated AD biomarkers may also help determine whether any clinical effect is accompanied by changes in disease-related biological pathways.

### 5.6. Limitations of the Current Evidence and Future Research Directions

Overall, the available evidence suggests that PEA-Lut can modulate inflammatory, oxidative, glial, and neurotrophic pathways across several experimental models. These findings support further investigation of PEA-Lut as a potential modulator of pathophysiological processes implicated in cerebrovascular and neurodegenerative diseases. Preclinical and preliminary clinical findings have been reported in TBI, stroke, PD, frontotemporal dementia, and AD. However, preclinical studies based on animal models may not fully recapitulate the multifactorial pathological process underlying neurodegenerative diseases, and human evidence remains limited and preliminary.

Some of the reported clinical studies are observational, uncontrolled, or non-randomized. In contrast, even the randomized placebo-controlled studies are mostly single-center studies conducted in small populations, with short longitudinal follow-up periods that limit assessment of potential long-term effects.

Furthermore, some trials were conducted by the same research groups, which may represent a potential source of bias in the reported findings.

The preclinical and clinical studies described in this review are summarized in the Supplementary Materials in Tables S1 and S2, respectively.

**Figure 2:**
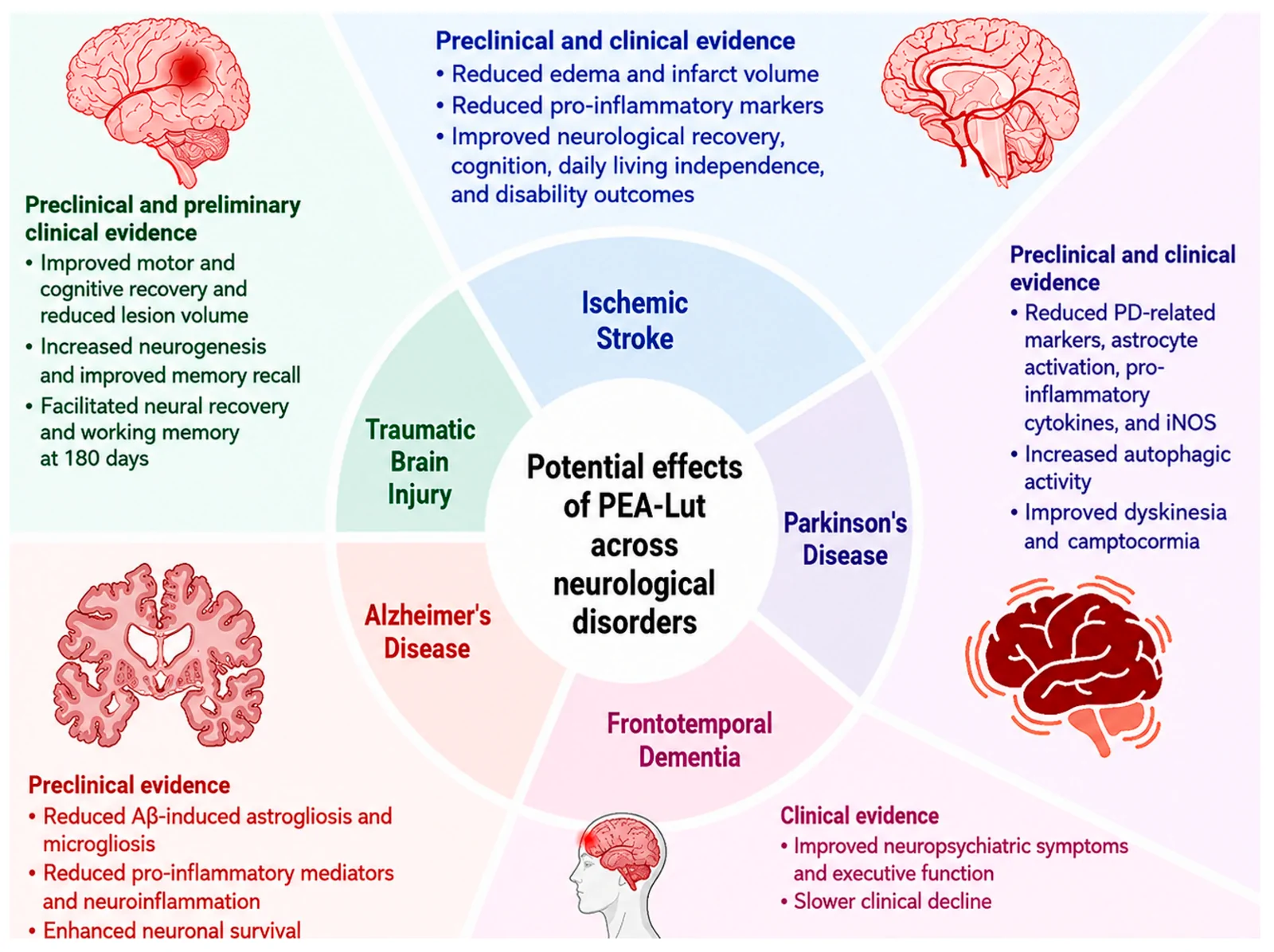
Proposed biological effects of co-ultramicronized PEA-Lut relevant to brain aging and cognitive impairment.

The current evidence therefore supports further clinical investigation but does not establish PEA-Lut as an effective intervention for preventing dementia or treating cognitive impairment. The main effects of PEA-Lut on brain aging and cognitive impairment are summarized in Figure 2.

The figure summarizes the potential effects of co-ultramicronized PEA-Lut across different neurological disorders, including ischemic stroke, TBI, AD, PD, and frontotemporal dementia. Available evidence suggests possible neuroprotective, anti-inflammatory, and neuromodulatory effects, with reported improvements in neurological, cognitive, behavioral, and functional outcomes across different experimental and clinical settings. However, the strength of evidence varies substantially among neurological disorders, with several findings deriving from preclinical models and a limited number of preliminary clinical studies. Therefore, the depicted effects should be considered potential rather than established therapeutic effects, and further well-designed clinical studies are needed to confirm their clinical relevance and efficacy.

## 6. Conclusions

The potential biological effects of co-ultramicronized PEA-Lut appear to extend beyond the modulation of neuroinflammation. The complementary properties of its two components—PEA acting mainly through the regulation of PPAR-α-related and other inflammatory pathways, and luteolin exerting antioxidant and anti-inflammatory effects—provide a rationale for their combined administration. Experimental evidence suggests that PEA-Lut may influence several interconnected mechanisms involved in brain aging and neurodegeneration, including chronic low-grade inflammation, oxidative and nitrosative stress, glial activation, mitochondrial dysfunction, and neurotrophic signaling.

These mechanisms are relevant because their interaction may contribute to the progressive loss of cellular homeostasis and increased vulnerability to age-related neurological disorders. In this context, PEA-Lut represents a biologically plausible strategy for further investigation. Its potential to influence multiple pathways may warrant investigation in older individuals, although the safety and pharmacological implications of its use in the context of multimorbidity and polypharmacy remain insufficiently characterized. The endogenous or naturally occurring origin of its components should not be interpreted as evidence of clinical efficacy or long-term safety.

Overall, the evidence supporting the use of PEA-Lut in neurodegenerative and cerebrovascular disorders remains insufficient for clinical translation. Although preclinical studies provide a biologically plausible rationale and preliminary clinical findings are encouraging in selected conditions, direct evidence in individuals with MCI due to AD or AD dementia is currently lacking. It therefore remains unclear whether PEA-Lut can prevent dementia, delay progression from MCI to AD, or improve clinically meaningful cognitive and functional outcomes.

Future studies should determine whether the timing of treatment influences the biological and clinical effects of Pea-Lut, particularly when administered during the early stages of cognitive impairment. This question should be addressed through adequately powered, multicenter, randomized, placebo-controlled trials with prespecified cognitive and functional outcomes, appropriate follow-up, and assessment of dose–response relationships, treatment duration, long-term safety, adherence, and potential interactions with standard therapies. Where possible, the inclusion of validated AD biomarkers would help determine whether any observed clinical effects are accompanied by changes in disease-related biological pathways. Until such evidence becomes available, PEA-Lut should be considered an investigational nutraceutical rather than an established intervention for cognitive impairment or dementia prevention.

## Data Availability

No new data were created or analyzed in this study. Data sharing is not applicable to this article.

## Supplementary Materials

The following supporting information can be downloaded at: https://www.mdpi.com/article/10.3390/nu18182949/s1, Table S1: Preclinical studies evaluating PEA and luteolin-based formulations in neurological disorders. Table S2: Clinical studies evaluating PEA and luteolin-based formulations in neurological disorders.

## References

[1] Gonzales M.M., Garbarino V.R., Pollet E., Palavicini J.P., Kellogg D.L., Kraig E., Orr M.E. (2022). Biological aging processes underlying cognitive decline and neurodegenerative disease. J. Clin. Investig..

[2] Davis M., O’Connell T., Johnson S., Cline S., Merikle E., Martenyi F., Simpson K. (2018). Estimating Alzheimer’s Disease Progression Rates from Normal Cognition Through Mild Cognitive Impairment and Stages of Dementia. Curr. Alzheimer Res..

[3] Grundman M., Petersen R.C., Ferris S.H., Thomas R.G., Aisen P.S., Bennett D.A., Foster N.L., Jack C.R., Galasko D.R., Doody R. (2004). Mild Cognitive Impairment Can Be Distinguished from Alzheimer Disease and Normal Aging for Clinical Trials. Arch. Neurol..

[4] Vega J.N., Newhouse P.A. (2014). Mild Cognitive Impairment: Diagnosis, Longitudinal Course, and Emerging Treatments. Curr. Psychiatry Rep..

[5] Castellani R.J., Rolston R.K., Smith M.A. (2010). Alzheimer Disease. Disease-a-Month.

[6] Lamptey R.N.L., Chaulagain B., Trivedi R., Gothwal A., Layek B., Singh J. (2022). A Review of the Common Neurodegenerative Disorders: Current Therapeutic Approaches and the Potential Role of Nanotherapeutics. Int. J. Mol. Sci..

[7] Vasic V., Barth K., Schmidt M.H.H. (2019). Neurodegeneration and Neuro-Regeneration—Alzheimer’s Disease and Stem Cell Therapy. Int. J. Mol. Sci..

[8] Punzi M., Sestieri C., Picerni E., Chiarelli A.M., Padulo C., Delli Pizzi A., Tullo M.G., Tosoni A., Granzotto A., Della Penna S. (2024). Atrophy of Hippocampal Subfields and Amygdala Nuclei in Subjects with Mild Cognitive Impairment Progressing to Alzheimer’s Disease. Heliyon.

[9] McDowell I. (2001). Alzheimer’s Disease: Insights from Epidemiology. Aging.

[10] Nichols E., Steinmetz J.D., Vollset S.E., Fukutaki K., Chalek J., Abd-Allah F., Abdoli A., Abualhasan A., Abu-Gharbieh E., Akram T.T. (2022). Estimation of the Global Prevalence of Dementia in 2019 and Forecasted Prevalence in 2050: An Analysis for the Global Burden of Disease Study 2019. Lancet Public Health.

[11] Ahmad M.A., Kareem O., Khushtar M., Akbar M., Haque M.R., Iqubal A., Haider M.F., Pottoo F.H., Abdulla F.S., Al-haidar M.B. (2022). Neuroinflammation: A Potential Risk for Dementia. Int. J. Mol. Sci..

[12] Luca M., Luca A., Calandra C. (2015). The Role of Oxidative Damage in the Pathogenesis and Progression of Alzheimer’s Disease and Vascular Dementia. Oxid. Med. Cell. Longev..

[13] Boccardi V., Pelini L., Ercolani S., Ruggiero C., Mecocci P. (2015). From Cellular Senescence to Alzheimer’s Disease: The Role of Telomere Shortening. Ageing Res. Rev..

[14] Ranson J.M., Rittman T., Hayat S., Brayne C., Jessen F., Blennow K., van Duijn C., Barkhof F., Tang E., Mummery C.J. (2021). Modifiable Risk Factors for Dementia and Dementia Risk Profiling. A User Manual for Brain Health Services-Part 2 of 6. Alzheimer’s Res. Ther..

[15] Veronese N., Soysal P., Demurtas J., Solmi M., Bruyère O., Christodoulou N., Ramalho R., Fusar-Poli P., Lappas A.S., Pinto D. (2023). Physical Activity and Exercise for the Prevention and Management of Mild Cognitive Impairment and Dementia: A Collaborative International Guideline. Eur. Geriatr. Med..

[16] Woods B., Rai H.K., Elliott E., Aguirre E., Orrell M., Spector A. (2023). Cognitive stimulation to improve cognitive functioning in people with dementia. Cochrane Database Syst. Rev..

[17] Dominguez L.J., Barbagallo M. (2018). Nutritional Prevention of Cognitive Decline and Dementia. Acta Biomed..

[18] Venetsanaki V., Pardali E.C., Cholevas C., Grammatikopoulou M.G., Goulis D.G., Theoharides T.C. (2026). Natural Molecules for Brain Health and Resilience. Int. J. Mol. Sci..

[19] Almatroodi S.A., Almatroudi A., Alharbi H.O.A., Khan A.A., Rahmani A.H. (2024). Effects and Mechanisms of Luteolin, a Plant-Based Flavonoid, in the Prevention of Cancers via Modulation of Inflammation and Cell Signaling Molecules. Molecules.

[20] López-Otín C., Blasco M.A., Partridge L., Serrano M., Kroemer G. (2013). The Hallmarks of Aging. Cell.

[21] López-Otín C., Blasco M.A., Partridge L., Serrano M., Kroemer G. (2023). Hallmarks of Aging: An Expanding Universe. Cell.

[22] Palomares D., Vanparys A.A.T., Jorgji J., Paître E., Kienlen-Campard P., Suelves N. (2025). Telomere-Driven Senescence Accelerates Tau Pathology, Neuroinflammation and Neurodegeneration in a Tauopathy Mouse Model. Acta Neuropathol. Commun..

[23] Hudson H.R., Sun X., Orr M.E. (2025). Senescent Brain Cell Types in Alzheimer’s Disease: Pathological Mechanisms and Therapeutic Opportunities. Neurotherapeutics.

[24] Ristori S., Bertoni G., Bientinesi E., Monti D. (2025). The Role of Nutraceuticals and Functional Foods in Mitigating Cellular Senescence and Its Related Aspects: A Key Strategy for Delaying or Preventing Aging and Neurodegenerative Disorders. Nutrients.

[25] Hoare M., Das T., Alexander G. (2010). Ageing, Telomeres, Senescence, and Liver Injury. J. Hepatol..

[26] Collado M., Blasco M.A., Serrano M. (2007). Cellular Senescence in Cancer and Aging. Cell.

[27] Roger L., Tomas F., Gire V. (2021). Mechanisms and Regulation of Cellular Senescence. Int. J. Mol. Sci..

[28] Herranz N., Gil J. (2018). Mechanisms and Functions of Cellular Senescence. J. Clin. Investig..

[29] Kurz D.J., Decary S., Hong Y., Erusalimsky J.D. (2000). Senescence-Associated β-Galactosidase Reflects an Increase in Lysosomal Mass during Replicative Ageing of Human Endothelial Cells. J. Cell Sci..

[30] Sweeney E.M., Abate G., Bakker B.R.V., Mastinu A., Lai Y., Uberti D., Nilsson P., Tambaro S. (2026). Cell Type-Specific Expression of P16, P21, and P53 Reveals Age-Dependent Glial Senescence in the AppNL-G-F Mouse Model of Alzheimer’s Disease. Aging Cell.

[31] Aird K.M., Zhang R. (2013). Detection of Senescence-Associated Heterochromatin Foci (SAHF). Methods Mol. Biol..

[32] Traa A., Keil A., AlOkda A., Jacob-Tomas S., Tamez González A.A., Zhu S., Rudich Z., Van Raamsdonk J.M. (2024). Overexpression of Mitochondrial Fission or Mitochondrial Fusion Genes Enhances Resilience and Extends Longevity. Aging Cell.

[33] Lopes-Paciencia S., Saint-Germain E., Rowell M.C., Ruiz A.F., Kalegari P., Ferbeyre G. (2019). The Senescence-Associated Secretory Phenotype and Its Regulation. Cytokine.

[34] Martínez-Cué C., Rueda N. (2020). Cellular Senescence in Neurodegenerative Diseases. Front. Cell. Neurosci..

[35] Verkhratsky A., Zorec R., Rodriguez-Arellano J.J., Parpura V. (2019). Neuroglia in Ageing. Adv. Exp. Med. Biol..

[36] Dougnon G., Matsui H. (2025). Lipofuscin Accumulation in Aging and Neurodegeneration: A Potential “Timebomb” Overlooked in Alzheimer’s Disease. Transl. Neurodegener..

[37] Salminen A., Ojala J., Kaarniranta K., Haapasalo A., Hiltunen M., Soininen H. (2011). Astrocytes in the Aging Brain Express Characteristics of Senescence-Associated Secretory Phenotype. Eur. J. Neurosci..

[38] Jiang T., Cadenas E. (2014). Astrocytic Metabolic and Inflammatory Changes as a Function of Age. Aging Cell.

[39] Lü L., Li J., Yew D.T., Rudd J.A., Mak Y.T. (2008). Oxidative Stress on the Astrocytes in Culture Derived from a Senescence Accelerated Mouse Strain. Neurochem. Int..

[40] Streit W.J., Xue Q.S., Tischer J., Bechmann I. (2014). Microglial Pathology. Acta Neuropathol. Commun..

[41] Yu H.M., Zhao Y.M., Luo X.G., Feng Y., Ren Y., Shang H., He Z.Y., Luo X.M., Chen S.D., Wang X.Y. (2012). Repeated lipopolysaccharide stimulation induces cellular senescence in BV2 cells. Neuroimmunomodulation.

[42] Narita M. (2007). Cellular Senescence and Chromatin Organisation. Br. J. Cancer.

[43] Tan X., Gao N. (2025). The Emerging Role of Cellular Senescence in Amyotrophic Lateral Sclerosis. Front. Neurosci..

[44] Baker D.J., Petersen R.C. (2018). Cellular Senescence in Brain Aging and Neurodegenerative Diseases: Evidence and Perspectives. J. Clin. Investig..

[45] Suryadevara V., Hudgins A.D., Rajesh A., Pappalardo A., Karpova A., Dey A.K., Hertzel A., Agudelo A., Rocha A., Soygur B. (2024). SenNet Recommendations for Detecting Senescent Cells in Different Tissues. Nat. Rev. Mol. Cell Biol..

[46] Tan F.C.C., Hutchison E.R., Eitan E., Mattson M.P. (2014). Are There Roles for Brain Cell Senescence in Aging and Neurodegenerative Disorders?. Biogerontology.

[47] Myung N.H., Zhu X., Kruman I.I., Castellani R.J., Petersen R.B., Siedlak S.L., Perry G., Smith M.A., Lee H.G. (2008). Evidence of DNA Damage in Alzheimer Disease: Phosphorylation of Histone H2AX in Astrocytes. Age.

[48] Panossian L.A., Porter V.R., Valenzuela H.F., Zhu X., Reback E., Masterman D., Cummings J.L., Effros R.B. (2003). Telomere Shortening in T Cells Correlates with Alzheimer’s Disease Status. Neurobiol. Aging.

[49] Chinta S.J., Woods G., Rane A., Demaria M., Campisi J., Andersen J.K. (2015). Cellular Senescence and the Aging Brain. Exp. Gerontol..

[50] Bronzuoli M.R., Facchinetti R., Valenza M., Cassano T., Steardo L., Scuderi C. (2019). Astrocyte Function Is Affected by Aging and Not Alzheimer’s Disease: A Preliminary Investigation in Hippocampi of 3xTg-AD Mice. Front. Pharmacol..

[51] Osellame L.D., Blacker T.S., Duchen M.R. (2012). Cellular and Molecular Mechanisms of Mitochondrial Function. Best Pract. Res. Clin. Endocrinol. Metab..

[52] Srivastava S. (2017). The Mitochondrial Basis of Aging and Age-Related Disorders. Genes.

[53] Tönnies E., Trushina E. (2017). Oxidative Stress, Synaptic Dysfunction, and Alzheimer’s Disease. J. Alzheimer’s Dis..

[54] Lee K.H., Cha M., Lee B.H. (2021). Crosstalk between Neuron and Glial Cells in Oxidative Injury and Neuroprotection. Int. J. Mol. Sci..

[55] Sun M.S., Jin H., Sun X., Huang S., Zhang F.L., Guo Z.N., Yang Y. (2018). Free Radical Damage in Ischemia-Reperfusion Injury: An Obstacle in Acute Ischemic Stroke after Revascularization Therapy. Oxid. Med. Cell. Longev..

[56] Robillard J.M., Gordon G.R., Choi H.B., Christie B.R., MacVicar B.A. (2011). Glutathione Restores the Mechanism of Synaptic Plasticity in Aged Mice to That of the Adult. PLoS ONE.

[57] Teleanu D.M., Niculescu A.G., Lungu I.I., Radu C.I., Vladâcenco O., Roza E., Costăchescu B., Grumezescu A.M., Teleanu R.I. (2022). An Overview of Oxidative Stress, Neuroinflammation, and Neurodegenerative Diseases. Int. J. Mol. Sci..

[58] Chen L., Deng H., Cui H., Fang J., Zuo Z., Deng J., Li Y., Wang X., Zhao L. (2017). Inflammatory Responses and Inflammation-Associated Diseases in Organs. Oncotarget.

[59] Chuang K.A., Li M.H., Lin N.H., Chang C.H., Lu I.H., Pan I.H., Takahashi T., Der Perng M., Wen S.F. (2017). Rhinacanthin C Alleviates Amyloid-β Fibrils’ Toxicity on Neurons and Attenuates Neuroinflammation Triggered by LPS, Amyloid-β, and Interferon-γ in Glial Cells. Oxid. Med. Cell. Longev..

[60] Cai Y., Liu J., Wang B., Sun M., Yang H. (2022). Microglia in the Neuroinflammatory Pathogenesis of Alzheimer’s Disease and Related Therapeutic Targets. Front. Immunol..

[61] Fruhwürth S., Zetterberg H., Paludan S.R. (2024). Microglia and Amyloid Plaque Formation in Alzheimer’s Disease—Evidence, Possible Mechanisms, and Future Challenges. J. Neuroimmunol..

[62] Leonardo S., Fregni F. (2023). Association of Inflammation and Cognition in the Elderly: A Systematic Review and Meta-Analysis. Front. Aging Neurosci..

[63] Bleve A., Motta F., Durante B., Pandolfo C., Selmi C., Sica A. (2023). Immunosenescence, Inflammaging, and Frailty: Role of Myeloid Cells in Age-Related Diseases. Clin. Rev. Allergy Immunol..

[64] Latham A.S., Moreno J.A., Geer C.E. (2023). Biological Agents and the Aging Brain: Glial Inflammation and Neurotoxic Signaling. Front. Aging.

[65] Kwon H.S., Koh S.H. (2020). Neuroinflammation in Neurodegenerative Disorders: The Roles of Microglia and Astrocytes. Transl. Neurodegener..

[66] Pawate S., Bhat N.R. (2008). Role of Glia in CNS Inflammation. Handbook of Neurochemistry and Molecular Neurobiology.

[67] Zhang Y.M., Qi Y.B., Gao Y.N., Chen W.G., Zhou T., Zang Y., Li J. (2023). Astrocyte Metabolism and Signaling Pathways in the CNS. Front. Neurosci..

[68] Valenza M., Facchinetti R., Menegoni G., Steardo L., Scuderi C. (2021). Alternative Targets to Fight Alzheimer’s Disease: Focus on Astrocytes. Biomolecules.

[69] Liu H.M., Cheng M.Y., Xun M.H., Zhao Z.W., Zhang Y., Tang W., Cheng J., Ni J., Wang W. (2023). Possible Mechanisms of Oxidative Stress-Induced Skin Cellular Senescence, Inflammation, and Cancer and the Therapeutic Potential of Plant Polyphenols. Int. J. Mol. Sci..

[70] Biswas S.K. (2016). Does the Interdependence between Oxidative Stress and Inflammation Explain the Antioxidant Paradox?. Oxid. Med. Cell. Longev..

[71] Han M., Wang N., Song M., Liu C., Wu Y., Yin J., Jin L., Jin W., Gao Z. (2026). The Mechanisms and Strategies for Alzheimer’s Disease Prevention: An Update. Acta Pharm. Sin. B.

[72] Zhang R., Zhang M., Wang P. (2025). The intricate interplay between dietary habits and cognitive function: Insights from the gut-brain axis. Front. Nutr..

[73] Kouvari M., D’cunha N.M., Travica N., Sergi D., Zec M., Marx W., Naumovski N. (2022). Metabolic Syndrome, Cognitive Impairment and the Role of Diet: A Narrative Review. Nutrients.

[74] Lee M.B., Hill C.M., Bitto A., Kaeberlein M. (2021). Anti-Aging Diets: Separating Fact from Fiction. Science.

[75] Brøns C., Jensen C.B., Storgaard H., Hiscock N.J., White A., Appel J.S., Jacobsen S., Nilsson E., Larsen C.M., Astrup A. (2009). Impact of Short-Term High-Fat Feeding on Glucose and Insulin Metabolism in Young Healthy Men. J. Physiol..

[76] Tian W., Cao S., Guan Y., Zhang Z., Liu Q., Ju J., Xi R., Bai R. (2025). The Effects of Low-Carbohydrate Diet on Glucose and Lipid Metabolism in Overweight or Obese Patients with T2DM: A Meta-Analysis of Randomized Controlled Trials. Front. Nutr..

[77] Puri S., Shaheen M., Grover B. (2023). Nutrition and Cognitive Health: A Life Course Approach. Front. Public Health.

[78] Liao Z.B., Hu Z.C., Zeng G.H., Chen J., Li X.P., Liu Y.H., Yao X.Q., Wang Y.R. (2026). The Association between Omega-3 Supplementation and Cognitive Decline in Older Adults. J. Prev. Alzheimer’s Dis..

[79] Buckinx F., Aubertin-Leheudre M. (2021). Nutrition to Prevent or Treat Cognitive Impairment in Older Adults: A GRADE Recommendation. J. Prev. Alzheimer’s Dis..

[80] Feng J., Zheng Y., Guo M., Ares I., Martínez M., Lopez-Torres B., Martínez-Larrañaga M.R., Wang X., Anadón A., Martínez M.A. (2023). Oxidative Stress, the Blood–Brain Barrier and Neurodegenerative Diseases: The Critical Beneficial Role of Dietary Antioxidants. Acta Pharm. Sin. B.

[81] Altınsoy C., Kahramanoğlu Aksoy E., Özgül S., Dikmen D. (2026). Nutritional Approaches to Managing Brain Fog: Insights Into Neuroinflammation, the Gut-Brain Axis, and Sleep. Curr. Nutr. Rep..

[82] La Fata G., Weber P., Mohajeri M.H. (2014). Effects of Vitamin E on Cognitive Performance during Ageing and in Alzheimer’s Disease. Nutrients.

[83] Fekete M., Lehoczki A., Tarantini S., Fazekas-Pongor V., Csípő T., Csizmadia Z., Varga J.T. (2023). Improving Cognitive Function with Nutritional Supplements in Aging: A Comprehensive Narrative Review of Clinical Studies Investigating the Effects of Vitamins, Minerals, Antioxidants, and Other Dietary Supplements. Nutrients.

[84] Beilharz J.E., Maniam J., Morris M.J. (2015). Diet-Induced Cognitive Deficits: The Role of Fat and Sugar, Potential Mechanisms and Nutritional Interventions. Nutrients.

[85] Flanagan E., Lamport D., Brennan L., Burnet P., Calabrese V., Cunnane S.C., de Wilde M.C., Dye L., Farrimond J.A., Emerson Lombardo N. (2020). Nutrition and the Ageing Brain: Moving towards Clinical Applications. Ageing Res. Rev..

[86] Sharma R., Padwad Y. (2020). Perspectives of the Potential Implications of Polyphenols in Influencing the Interrelationship between Oxi-Inflammatory Stress, Cellular Senescence and Immunosenescence during Aging. Trends Food Sci. Technol..

[87] Santini A., Tenore G.C., Novellino E. (2017). Nutraceuticals: A Paradigm of Proactive Medicine. Eur. J. Pharm. Sci..

[88] Caponio G.R., Lippolis T., Tutino V., Gigante I., De Nunzio V., Milella R.A., Gasparro M., Notarnicola M. (2022). Nutraceuticals: Focus on Anti-Inflammatory, Anti-Cancer, Antioxidant Properties in Gastrointestinal Tract. Antioxidants.

[89] Nasri H., Baradaran A., Shirzad H., Kopaei M.R. (2014). New Concepts in Nutraceuticals as Alternative for Pharmaceuticals. Int. J. Prev. Med..

[90] Mecocci P., Tinarelli C., Schulz R.J., Polidori M.C. (2014). Nutraceuticals in Cognitive Impairment and Alzheimer’s Disease. Front. Pharmacol..

[91] Mentella M.C., Scaldaferri F., Ricci C., Gasbarrini A., Miggiano G.A.D. (2019). Cancer and Mediterranean Diet: A Review. Nutrients.

[92] Reza-Zaldívar E.E., Jacobo-Velázquez D.A. (2023). Comprehensive Review of Nutraceuticals against Cognitive Decline Associated with Alzheimer’s Disease. ACS Omega.

[93] Wei B.Z., Li L., Dong C.W., Tan C.C., Xu W. (2023). The Relationship of Omega-3 Fatty Acids with Dementia and Cognitive Decline: Evidence from Prospective Cohort Studies of Supplementation, Dietary Intake, and Blood Markers. Am. J. Clin. Nutr..

[94] Wang Z., Zhu W., Xing Y., Jia J., Tang Y. (2022). B Vitamins and Prevention of Cognitive Decline and Incident Dementia: A Systematic Review and Meta-Analysis. Nutr. Rev..

[95] Albadrani H.M., Chauhan P., Ashique S., Babu M.A., Iqbal D., Almutary A.G., Abomughaid M.M., Kamal M., Paiva-Santos A.C., Alsaweed M. (2024). Mechanistic Insights into the Potential Role of Dietary Polyphenols and Their Nanoformulation in the Management of Alzheimer’s Disease. Biomed. Pharmacother..

[96] Wang L., Zhao T., Zhu X., Jiang Q. (2023). Low Blood Carotenoid Status in Dementia and Mild Cognitive Impairment: A Systematic Review and Meta-Analysis. BMC Geriatr..

[97] Zhou F., Xie X., Zhang H., Liu T. (2023). Effect of Antioxidant Intake Patterns on Risks of Dementia and Cognitive Decline. Eur. Geriatr. Med..

[98] Di Stefano V., Steardo L., D’Angelo M., Monaco F., Steardo L. (2025). Palmitoylethanolamide: A Multifunctional Molecule for Neuroprotection, Chronic Pain, and Immune Modulation. Biomedicines.

[99] Peritore A.F., Siracusa R., Crupi R., Cuzzocrea S. (2019). Therapeutic Efficacy of Palmitoylethanolamide and Its New Formulations in Synergy with Different Antioxidant Molecules Present in Diets. Nutrients.

[100] Clayton P., Hill M., Bogoda N., Subah S., Venkatesh R. (2021). Palmitoylethanolamide: A Natural Compound for Health Management. Int. J. Mol. Sci..

[101] Impellizzeri D., Bruschetta G., Cordaro M., Crupi R., Siracusa R., Esposito E., Cuzzocrea S. (2014). Micronized/Ultramicronized Palmitoylethanolamide Displays Superior Oral Efficacy Compared to Nonmicronized Palmitoylethanolamide in a Rat Model of Inflammatory Pain. J. Neuroinflamm..

[102] Cordaro M., Cuzzocrea S., Crupi R. (2020). An Update of Palmitoylethanolamide and Luteolin Effects in Preclinical and Clinical Studies of Neuroinflammatory Events. Antioxidants.

[103] Nabavi S.F., Braidy N., Gortzi O., Sobarzo-Sanchez E., Daglia M., Skalicka-Woźniak K., Nabavi S.M. (2015). Luteolin as an Anti-Inflammatory and Neuroprotective Agent: A Brief Review. Brain Res. Bull..

[104] Valenza M., Facchinetti R., Steardo L., Scuderi C. (2022). Palmitoylethanolamide and White Matter Lesions: Evidence for Therapeutic Implications. Biomolecules.

[105] Ntalouka F., Tsirivakou A. (2023). Luteolin: A Promising Natural Agent in Management of Pain in Chronic Conditions. Front. Pain Res..

[106] Petrosino S., Cordaro M., Verde R., Moriello A.S., Marcolongo G., Schievano C., Siracusa R., Piscitelli F., Peritore A.F., Crupi R. (2018). Oral Ultramicronized Palmitoylethanolamide: Plasma and Tissue Levels and Spinal Anti-Hyperalgesic Effect. Front. Pharmacol..

[107] Gabrielsson L., Mattsson S., Fowler C.J. (2016). Palmitoylethanolamide for the Treatment of Pain: Pharmacokinetics, Safety and Efficacy. Br. J. Clin. Pharmacol..

[108] Artamonov M., Zhukov O., Shuba I., Storozhuk L., Khmel T., Klimashevsky V., Mikosha A., Gula N. (2005). Incorporation of Labelled N-Acylethanolamine (NAE) into Rat Brain Regions in Vivo and Adaptive Properties of Saturated NAE under x-Ray Irradiation. Ukr. Biokhim. Zh. (1999).

[109] Mehkri S., Dinesh K.G., Ashok G., Bopanna K. (2025). Pharmacokinetics of Micronized and Water Dispersible Palmitoylethanolamide in Comparison with Standard Palmitoylethanolamide Following Single Oral Administration in Male Sprague-Dawley Rats. Indian J. Pharmacol..

[110] Pickering E., Steels E.L., Steadman K.J., Rao A., Vitetta L. (2022). A Randomized Controlled Trial Assessing the Safety and Efficacy of Palmitoylethanolamide for Treating Diabetic-Related Peripheral Neuropathic Pain. Inflammopharmacology.

[111] Briskey D., Skinner R., Smith C., Rao A. (2024). Effectiveness of Palmitoylethanolamide (Levagen+) Compared to a Placebo for Reducing Pain, Duration, and Medication Use during Migraines in Otherwise Healthy Participants-A Double-Blind Randomised Controlled Study. Pharmaceuticals.

[112] Assogna M., Di Lorenzo F., Bonnì S., Borghi I., Cerulli Irelli E., Mencarelli L., Maiella M., Minei M., Esposito R., Casula E.P. (2025). Phase 2 Study of Palmitoylethanolamide Combined with Luteoline in Frontotemporal Dementia Patients. Brain Commun..

[113] Di Stadio A., D’Ascanio L., Vaira L.A., Cantone E., De Luca P., Cingolani C., Motta G., De Riu G., Vitelli F., Spriano G. (2022). Ultramicronized Palmitoylethanolamide and Luteolin Supplement Combined with Olfactory Training to Treat Post-COVID-19 Olfactory Impairment: A Multi-Center Double-Blinded Randomized Placebo-Controlled Clinical Trial. Curr. Neuropharmacol..

[114] Bonzanino M., Riolo M., Battaglini I., Perna M., De Mattei M. (2024). PEALut in the Dietary Management of Patients with Acute Ischemic Stroke: A Prospective Randomized Controlled Clinical Trial. J. Clin. Med..

[115] Bortoletto R., Comacchio C., Garzitto M., Piscitelli F., Balestrieri M., Colizzi M. (2025). Palmitoylethanolamide Supplementation for Human Health: A State-of-the-Art Systematic Review of Randomized Controlled Trials in Patient Populations. Brain Behav. Immun. Health.

[116] Lang-Illievich K., Klivinyi C., Lasser C., Brenna C.T.A., Szilagyi I.S., Bornemann-Cimenti H. (2023). Palmitoylethanolamide in the Treatment of Chronic Pain: A Systematic Review and Meta-Analysis of Double-Blind Randomized Controlled Trials. Nutrients.

[117] Petrosino S., Moriello A.S. (2020). Palmitoylethanolamide: A Nutritional Approach to Keep Neuroinflammation within Physiological Boundaries-A Systematic Review. Int. J. Mol. Sci..

[118] Scuderi C., Valenza M., Stecca C., Esposito G., Carratù M.R., Steardo L. (2012). Palmitoylethanolamide Exerts Neuroprotective Effects in Mixed Neuroglial Cultures and Organotypic Hippocampal Slices via Peroxisome Proliferator-Activated Receptor-α. J. Neuroinflamm..

[119] Cordaro M., Impellizzeri D., Paterniti I., Bruschetta G., Siracusa R., De Stefano D., Cuzzocrea S., Esposito E. (2016). Neuroprotective Effects of Co-UltraPEALut on Secondary Inflammatory Process and Autophagy Involved in Traumatic Brain Injury. J. Neurotrauma.

[120] Campolo M., Crupi R., Cordaro M., Cardali S.M., Ardizzone A., Casili G., Scuderi S.A., Siracusa R., Esposito E., Conti A. (2021). Co-Ultra PEALut Enhances Endogenous Repair Response Following Moderate Traumatic Brain Injury. Int. J. Mol. Sci..

[121] Caltagirone C., Cisari C., Schievano C., Di Paola R., Cordaro M., Bruschetta G., Esposito E., Cuzzocrea S., Ventura F., Casaleggio M. (2016). Co-Ultramicronized Palmitoylethanolamide/Luteolin in the Treatment of Cerebral Ischemia: From Rodent to Man. Transl. Stroke Res..

[122] Siracusa R., Paterniti I., Impellizzeri D., Cordaro M., Crupi R., Navarra M., Cuzzocrea S., Esposito E. (2015). The Association of Palmitoylethanolamide with Luteolin Decreases Neuroinflammation and Stimulates Autophagy in Parkinson’s Disease Model. CNS Neurol. Disord. Drug Targets.

[123] Brotini S. (2021). Palmitoylethanolamide/Luteolin as Adjuvant Therapy to Improve an Unusual Case of Camptocormia in a Patient with Parkinson’s Disease: A Case Report. Innov. Clin. Neurosci..

[124] Assogna M., Casula E.P., Borghi I., Bonnì S., Samà D., Motta C., Di Lorenzo F., D’Acunto A., Porrazzini F., Minei M. (2020). Effects of Palmitoylethanolamide Combined with Luteoline on Frontal Lobe Functions, High Frequency Oscillations, and GABAergic Transmission in Patients with Frontotemporal Dementia. J. Alzheimer’s Dis..

[125] Paterniti I., Cordaro M., Campolo M., Siracusa R., Cornelius C., Navarra M., Cuzzocrea S., Esposito E. (2014). Neuroprotection by Association of Palmitoylethanolamide with Luteolin in Experimental Alzheimer’s Disease Models: The Control of Neuroinflammation. CNS Neurol. Disord. Drug Targets.

[126] Facchinetti R., Valenza M., Bronzuoli M.R., Menegoni G., Ratano P., Steardo L., Campolongo P., Scuderi C. (2020). Looking for a Treatment for the Early Stage of Alzheimer’s Disease: Preclinical Evidence with Co-Ultramicronized Palmitoylethanolamide and Luteolin. Int. J. Mol. Sci..

[127] Facchinetti R., Valenza M., Gomiero C., Mancini G.F., Steardo L., Campolongo P., Scuderi C. (2022). Co-Ultramicronized Palmitoylethanolamide/Luteolin Restores Oligodendrocyte Homeostasis via Peroxisome Proliferator-Activated Receptor-α in an In Vitro Model of Alzheimer’s Disease. Biomedicines.

[128] Facchinetti R., Valenza M., Ciarla C., Steardo L., Serviddio G., Palombelli G., Verkhratsky A., Steardo L., Canese R., Cassano T. (2026). Ultramicronized Palmitoylethanolamide Restores Astrocyte-Neuron Metabolic Coupling and Klotho/FGF21 Signaling in a Triple-Transgenic Mouse Model of Alzheimer’s Disease. Biomed. Pharmacother..

