# Supplementary material for: Palmitoylethanolamide and Luteolin in Brain Aging and Cognitive Decline: Biological Rationale and Current Evidence
Source: Nutrients. 2026 Sep 9;18(18):2949. doi: 10.3390/nu18182949 (PMC13610771; doi:10.3390/nu18182949)
Supplement: Supplementary file 1 [file nutrients-18-02949-s001.zip › nutrients-4486999-supplementary.pdf]

Table S1. Preclinical studies evaluating PEA and luteolin-based formulations in neurological disorders.

Preclinical study				
**Author, year**	**Study type and population**	**Intervention**	**Main results**	**Limitations**
**Cordaro et al., 2016 [119]**	Preclinical mouse TBI model.	*Kind of product:* Co-ultraPEA-Lut *Dose*: 1 mg/kg/day for 72 hours.	Improved motor and cognitive recovery and reduced lesion volume.	Limited translation relevance; limited assessment of long-term functional outcomes.
**Campolo et al., 2021 [120]**	Preclinical mouse TBI model.	*Kind of product:* Co-ultraPEA-Lut *Dose*: 1 mg/kg for 72 hours or 7 days.	Increased neurogenesis at 72 h and 7 days after TBI, with improved memory recall.	Limited translation validity.
**Caltagirone et al., 2016 [121]**	Preclinical rat model of cerebral ischemia (middle cerebral artery occlusion)	*Kind of product:* Co-ultraPEA-Lut *Dose*: 1 mg/kg 1 h after ischemia and 6 h after reperfusion	Reduced brain edema and infarct volume; improved neurobehavioral function; reduced pro-inflammatory and astrocytic markers.	Animal model; acute treatment; limited translational relevance.
**Siracusa et al., 2015 [122]**	Preclinical study in a mouse PD model.	*Kind of product:* Co-ultraPEA-Lut *Dose*: 1 mg/kg/day, for 7 days	Reduced neuroinflammation and oxidative stress; increased autophagy and neuroprotection.	Preclinical model; short treatment period; limited translational relevance.
**Paterniti et al., 2014 [125]**	Preclinical study in in vitro and ex vivo models of AD.	*Kind of product:* Co-ultraPEA-Lut *Dose*: 0.27–27 μM PEA	Reduced neuroinflammation and apoptosis; preserved neuronal and neurotrophic markers.	In vitro/ex vivo models; no in vivo validation; limited translational relevance.
**Facchinetti et al., 2020 [126]**	Preclinical mouse AD model.	*Kind of product:* Co-ultraPEA-Lut *Dose:* 5 mg/kg/day, for 14 days	Reduced astrogliosis, microgliosis, neuroinflammation, and neuronal damage; preserved BDNF and GDNF expression.	Animal model; short treatment duration; limited translational relevance.
**Facchinetti et al., 2022 [127]**	Preclinical study in an in vitro model of AD.	*Kind of product:* Co-ultraPEA-Lut *Dose:* 3 μM, for 48 h	Reduced Aβ-induced astrocyte reactivity and inflammation; preserved oligodendrocyte morphology and maturation	In vitro model; absence of neurons; limited translational relevance
**Facchinetti et al., 2026 [128]**	Preclinical mouse AD model.	*Kind of product:* Ultramicronized PEA *Dose*: 28 mg, for 90 days.	Restored lactate–glutamate and taurine metabolism and increased FGF21/Klotho signaling	Animal model; small sample size; limited translational relevance

Abbreviations: AD, Alzheimer's disease; Co-ultraPEALut, co-ultramicronized palmitoylethanolamide/luteolin; Lut, luteolin; PEA, palmitoylethanolamide; PD, Parkinson's disease; TBI, traumatic brain injury.

Table S2. Clinical studies evaluating PEA and luteolin-based formulations in neurological disorders.

Clinical study				
**Author, year**	**Study type and population**	**Intervention**	**Main results**	**Limitations**
**Campolo et al., 2021 [120]**	Randomized controlled clinical trial in patients with moderate TBI.	*Kind of product:* Co-ultraPEALut<br>*Dose:* 700 mg PEA + 70 mg Lut, bis/die for 180 days.<br>*Sample Size:* 30 completed.	Preliminary findings at 180 days suggest a potential improvement in working memory.	Small sample size and single-center design.<br>Potential confounding by spontaneous recovery.
**Caltagirone et al., 2016 [121]**	Observational clinical study in patients with ischemic stroke undergoing neurorehabilitation.	*Kind of product:* Co-ultraPEALut<br>*Dose:* 700 mg PEA + 70 mg Lut, bis/die for 60 days.<br>*Sample Size:* 250 patients.	Improved neurological status, cognition, spasticity, pain, and ADL independence after 60 days.	Observational, uncontrolled study; no randomized comparator; potential effects of rehabilitation and spontaneous recovery
**Bonzanino et al., 2024 [114]**	Prospective, randomized, open-label controlled clinical trial in patients with acute ischemic stroke undergoing thrombolysis.	*Kind of product:* Co-ultraPEALut<br>*Dose:* 700 mg PEA + 70 mg Lut, bis/die for 90 days.<br>*Sample Size:* 60 patients (30 PEA-Lut and 30 control).	Improved neurological, functional, disability, and cognitive outcomes vs. standard care.	Small sample, single-center, open-label and short follow-up.
**Brotini S., 2021 [123]**	Case report in a patient with PD.	*Kind of product:* Co-ultraPEALut<br>*Dose:* 700 mg PEA + 70 mg Lut, bis/die.	Improved dyskinesia and reduced camptocormia; complete resolution of leg and trunk dyskinesia after 4 months	Single-patient case report; no control group; concomitant dopaminergic therapy; limited generalizability.
**Assogna et al., 2020 [124]**	Pilot study in patients with FTD.	*Kind of product:* PEA-Lut<br>*Dose:* 700 mg bis/die for 4 weeks.<br>*Sample Size:* 17 patients.	Improved behavioral and executive function; restored GABAergic activity and increased frontal beta/gamma oscillations.	Small sample;<br>no placebo control;<br>short follow-up.
**Assogna et al., 2025 [112]**	Phase 2 randomized, double-blind, placebo-controlled clinical trial in patients with FTD.	*Kind of product:* Co-ultraPEALut<br>*Dose:* 700 mg PEA + 70 mg Lut, bis/die for 24 weeks.<br>*Sample Size:* 48 patients with probable FTD (25 treatment, 23 placebo)	Slower global, functional, and language decline compared with placebo.	Small, monocentric study;<br>short follow-up.

Abbreviations: ADL, activities of daily living; Co-ultraPEALut, co-ultramicronized palmitoylethanolamide/luteolin; FTD, frontotemporal dementia; Lut, luteolin; PEA, palmitoylethanolamide; TBI, traumatic brain injury.
